# Genes regulated by the *Escherichia coli *SOS repressor LexA exhibit heterogenous expression

**DOI:** 10.1186/1471-2180-10-283

**Published:** 2010-11-11

**Authors:** Simona Kamenšek, Zdravko Podlesek, Osnat Gillor, Darja Žgur-Bertok

**Affiliations:** 1 Dept. of Biology, Biotechnical Faculty, University of Ljubljana, Večna pot 111, Slovenia; 2 Zuckerberg Institute for Water Research, J. Blaustein Institutes for Desert Research, Ben-Gurion University, Israel

## Abstract

**Background:**

Phenotypic heterogeneity may ensure that a small fraction of a population survives environmental perturbations or may result in lysis in a subpopulation, to increase the survival of siblings. Genes involved in DNA repair and population dynamics play key roles in rapid responses to environmental conditions. In *Escherichia coli *the transcriptional repressor LexA controls a coordinated cellular response to DNA damage designated the SOS response. Expression of LexA regulated genes, e.g. colicin encoding genes, *recA*, *lexA *and *umuDC*, was examined utilizing transcription fusions with the promoterless *gfp *at the single cell level.

**Results:**

The investigated LexA regulated genes exhibited heterogeneity, as only in a small fraction of the population more intense fluorescence was observed. Unlike *recA *and *lexA*, the pore forming and nuclease colicin activity genes as well as *umuDC*, exhibited no basal level activity. However, in a *lexA *defective strain high level expression of the gene fusions was observed in the large majority of the cells. All of the investigated genes were expressed in a *recA *defective strain, albeit at lower levels, revealing expression in the absence of a spontaneous SOS response. In addition, the simultaneous expression of *cka*, encoding the pore forming colicin K, and *lexA*, investigated at the single cell level revealed high level expression of only *cka *in rare individual cells.

**Conclusion:**

LexA regulated genes exhibit phenotypic heterogeneity as high level expression is observed in only a small subpopulation of cells. Heterogenous expression is established primarily by stochastic factors and the binding affinity of LexA to SOS boxes.

## Background

Genetically identical bacterial cells can exhibit heterogeneity as the population bifurcates into distinct subpopulations. Such heterogeneity within clonal populations is a bet hedging strategy as a small fraction of a population is either prepared to survive adverse environmental conditions or sacrifice itself to enhance the likelihood of survival of clonal siblings. Examples of phenotypic heterogeneity include: development of competence and sporulation in *Bacillus subtilis*, lysogenic versus the lytic cycle of bacteriophage lambda, biofilm formation, toxin production and antibiotic persistence [1-4].

In *Escherichia coli *DNA damage induces the expression of more than 40 genes leading to arrest of cell division and the induction of DNA repair, prophages, toxin production and mutagenesis [5]. The key regulator of this coordinated cellular response, named the SOS response [6], is the LexA protein that represses gene expression by binding as a dimer to a 16-mer consensus sequence CTG-N_10_-CAG designated as SOS boxes [7]. Upon DNA damage replication forks are stalled exposing single-stranded DNA (ssDNA). RecA binds to the ssDNA forming a nucleoprotein filament which activates the autoproteolytic cleavage of LexA, leading to induction of the SOS response. In addition to activation by exogenous DNA damaging agents, the SOS response is also induced by endogenous, as well as spontaneous events [5].

SOS induction often results in the production of antimicrobial toxins such as bacteriocins. The bacteriocins of *E. coli *strains, designated as colicins, are plasmid encoded and are found with high frequency among natural isolates [8]. These toxins were suggested to promote phenotypic and genotypic diversity within *E. coli *populations in the mammalian colon [9,10]. Colicins destroy cells by one of three mechanisms: (i) they either form pores in the cytoplasmic membrane thus depleting its electrochemical potential, (ii) degrade either the DNA or RNA of their target cell or (iii) inhibit peptidoglycan and lipopolysaccharide (LPS) O-antigen biosynthesis [11-13]. Production and release of most colicins is encoded by three genes, an activity gene encoding the colicin protein, an immunity gene encoding a protein that protects the cell from its produced toxin and a lysis gene for semispecific release of the colicin. Colicin encoding genes characteristically have two overlapping SOS boxes that bind two LexA dimers and protect the cell from untimely colicin production, as it is lethal to the producing cell [14]. Some colicins, such as colicins B and M, have no lysis genes and are actively secreted by an unknown mechanism [15]. Colicin B and M encoding operons are tightly linked on large conjugative plasmids [16,17]. Expression of both colicin B and colicin M seems to be regulated by a common SOS boxes located upstream of the colicin B activity gene [16,18].

In previous studies we showed that the pore forming colicin K activity gene *cka *is expressed in only a small fraction of a bacterial population while the immunity gene encoding the immunity protein is expressed in the large majority of the cells [3,19]. In the present study we investigated, at the single cell level, expression of the activity genes of several other colicins namely, the pore formers A, E1 and N, the DNase colicin E7 and the LPS synthesis inhibitor, colicin M. We compared the single cell colicin expression to the expression of other LexA regulated genes, e.g. *recA*, *lexA *and *umuDC*, and finally we examined the simultaneous expression of the colicin encoding *cka *gene and the *lexA *gene.

## Methods

### Bacterial strains, plasmids and growth conditions

The bacterial strains and plasmids used in this study are presented in Table 1. Bacteria were grown in Luria-Bertani (LB) with aeration at 37°C and with the appropriate antibiotics. Ampicillin and kanamycin (Sigma, St Louis MO, USA) were used at concentrations 100 μg ml^-1 ^and 30 μg l^-1^, respectively.

**Table 1:** *E. coli *strains and plasmids used in the presented study

Bacterial strains	Relevant properties	Source/reference
RW118	*thr-1 araD139 *Δ*(gpt-proA)62 lacY1 tsx-33 supE44 galK2 hisG4 rpsL31 xyl-5 mtl-1 argE thi-1 sulA211*	R. Woodgate

RW464	RW118 *recA*	R. Woodgate

RW542	RW118 *lexA51 *(Def)	R. Woodgate

Plasmids		

pSC101 derivative	pSC101 low copy plasmid origin with promoterless *GFPmut3 *gene, Kn^r^	21

pSC300	*caa-gfp *Kn^r^	This study

pSC301	*cna-gfp *Kn^r^	This study

pSC302	*ce1a-gfp *Kn^r^	This study

pSC303	*ce7a-gfp *Kn^r^	This study

pSC304	*cma-gfp *Kn^r^	This study

pColA-CA31	*caa cai cal*	A. P. Pugsley

pColN-284	*cna cni cnl*	A. P. Pugsley

pColE1-K53	*ce1a ce1i ce1l*	A. P. Pugsley

pColE7-K317	*ce7a ce7i ce7l*	A. P. Pugsley

pCHAP1	*cma*	A. P. Pugsley

pSC200	*lexA-gfp *Kn^r^	21

pSC201	*recA-gfp *Kn^r^	21

pSC202	*umuD-gfp *Kn^r^	21

pSC203	*uvrA-gfp *Kn^r^	21

pDsRed-Express2-N1	*DsRed-Expre*ss2 reporter Kn^r^	B. Glick

pKCT3	*cka-gfp *Ap^r ^Kn^r^	19

pKCT10	*cka-DsRed-Express2 *Ap^r^	This study

### General DNA techniques

Plasmid DNA isolation was performed with the GeneJET™ plasmid miniprep kit (Fermentas, Burlington, Canada). Standard procedures were used for gel electrophoresis, ligations and transformation experiments [20]. Restriction endonuclease digestion was performed according to the instructions of the manufacturer (Fermentas). The PCR amplified fragments were purified using the QIAquick PCR purification kit (Qiagen, Hamburg, Germany). DNA fragments were isolated from agarose gels by using a QIAquick gel extraction kit (Qiagen).

### Construction of promoter fusions

PCR was carried out to amplify the promoter regions with an additional 73 - 93 bp of the flanking colicin encoding gene for colicins A (486 bp), E1 (508 bp), E7 (501 bp), N (499 bp) and M (298 bp) with the primers listed in Table 2. All primers have added *Bam*HI and *Xho*I restriction sites. The PCR generated fragments were cut with *Bam*HI and *Xho*I (Fermentas), and ligated into the low copy number pSC101 [21] based plasmid with a promoterless *(GFPmut3) gfp *also cut with the same two enzymes.

**Table 2:** Primers used in this study

Primers	nucleotide sequence 5'-3'
ColA-F	TCCTCGAGATGCTCTGATCAGTTCACT

ColA-R	TCGGATCCTACCACCACCCGGCTC

ColN-F	TCCTCGAGGATCAGTTCACTGGTTTCA

ColN-R	TCGGATCCGCCACTGGTATTACCAATG

ColE1-F	TCCTCGAGCAGTTCACTGGTTTCAACC

ColE1-R	TCGGATCCCCCGTCAGGAGTACCATTC

ColE7-F	TCCTCGAGAGGAATACAACACCTTAAA

ColE7-R	TCGGATCCTAGGGCCGCCATTAATGTT

ColM-F	TCCTCGAGGAGTTCTCAATATATATTTCCAGT

ColM-R	TCGGATCCCAGGAACATGCGGTGCTGAA

The promoterless *DsRed-Express2 *gene, which is part of a gene cassette on plasmid pDsRed-Express2-N1, was cloned into the natural colicin K encoding plasmid pColK-K235 manipulated to carry the Ap^r ^gene as a selectable marker, and a *Kpn*I restriction site in the *cka *gene. A cassette carrying the promoterless *gfp *was inserted at the *KpnI *restriction site [19]. The *cka-DsRed-Express2 *transcriptional fusion was constructed by replacing the *Kpn*I fragment harboring the promoterless *gfp *with the in frame promoterless *DsRed-Express2 *[22] amplified by PCR (Table 2) which was prior to ligation also cut with *Kpn*I. Only cells with an active *cka *promoter can express DsRed-Express2.

Nucleotide sequencing was performed to confirm that no base changes had occurred during amplification. The sequences have been deposited in the GenBank Nucleotide sequence database under accession numbers, HM449002 (*caa *promoter region), HM449003 (*cna *promoter region), HM449004 (*ce1a *promoter region), HM449005 (*ce7a *promoter region), HM449006 (*cma *promoter region).

### Fluorescence microscopy

Strains RW118 and RW464 carrying different colicin promoter region-*gfp *transcriptional fusions, and control strains without plasmid carrying *gfp *fusions, were grown with aeration at 37°C. Samples were removed at early stationary phase and chloramphenicol (500 μg ml^-1^) (Sigma) was added to block protein synthesis. Prior to microscopy, cells were attached to glass slides coated with 0.1% (wt vol^-1^) poly-L-lysine (Sigma). Fluorescence microscopy to detect expression in single cells was performed using an inverted microscope (Nikon Eclipse TE300), equipped with a Nikon digital camera DXM 1200, and a 488 nm Argon-Ion laser as well as bright field microscopy.

The examined cells were counted with software for quantification of bacteria by automated image analysis cellC http://www.cs.tut.fi/sgn/csb/cellc/. The fluorescence intensity of individual cells was estimated using image analysis software Scion Image http://www.scioncorp.com as previously described [3]. The fluorescent micrographs were converted to greyscale images. The density window was established by using density slice matching the shape of the cells with the highest fluorescence intensity and that of the cells with the lowest intensity, gaining the top and the bottom boundaries (respectively) of the density window. For greater clearness the density index scale is determined from 0 (black) to 256 (white). All micrographs were taken at exactly the same conditions; thus the density window gives good correlation to the fluorescence intensity of the analyzed population.

Simultaneous expression of the *cka-DsRed-Express2 *and the *lexA-gfp *fusions was investigated employing a laser scanning Confocal Microscope (Zeiss, Göttingen, Germany).

## Results and discussion

### Pore forming and nuclease colicins exhibit heterogeneity

**Figure 1:**
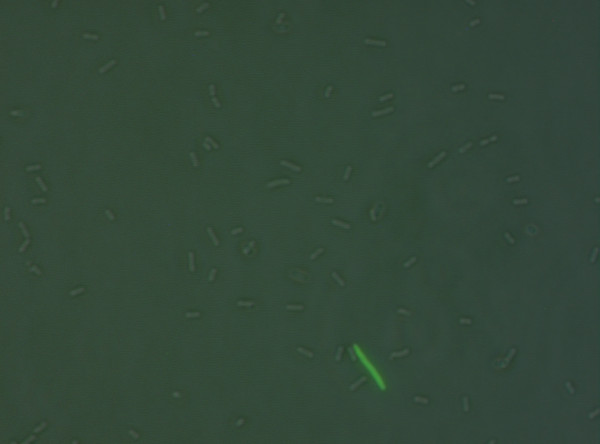
**Merged image of the phase contrast and fluorescence images of RW118 with a *caa-gfp *transcriptional fusion**. Only a small subpopulation of cells exhibited high fluorescence intensity, while the large majority of the cells exhibited no fluorescence.

**Figure 2:**
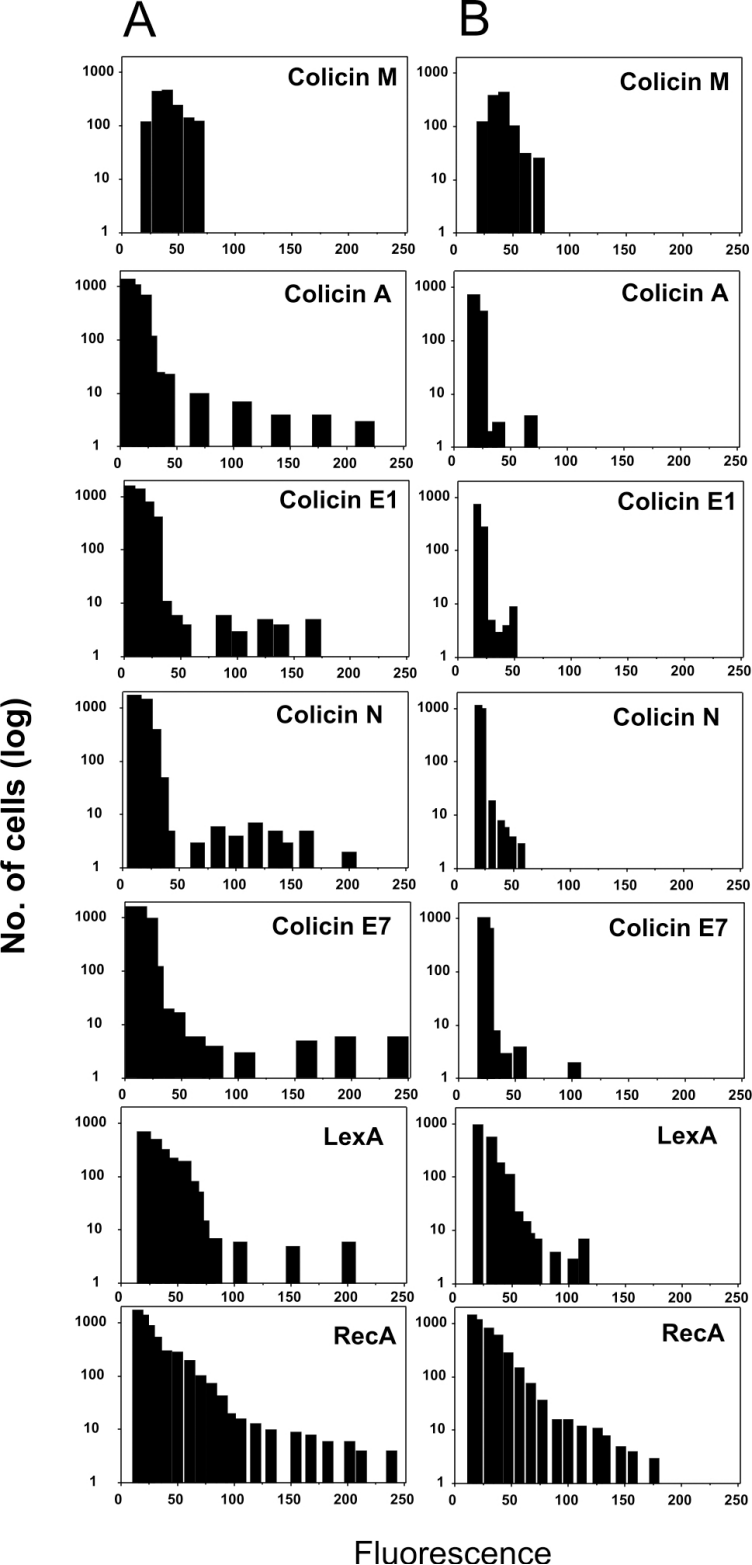
**Quantification of fluorescence intensity among strains expressing *gfp *transcriptional fusions**. Number of cells from digital micrographs were calculated and to each cell the relative fluorescence was assigned with the use of Scion Image software. The average fluorescence value and number of cells within a narrow interval was plotted. **A: **Expression of *gfp *transcriptional fusions in RW118 and **B: **Expression in isogenic *recA *defective RW464.

The advent of methods for visualization of gene expression in individual cells has revealed within populations of genetically identical bacteria heterogeneity in expression of certain genes [1-3]. A classical example of heterogeneity is the expression of the *cka *gene, encoding the pore forming colicin K; in the absence of exogenous DNA damaging agents *cka *is expressed in only a small fraction of the population [3,19] as the producing cells lyse to release the colicin. While colicin expression is characteristically regulated by the LexA protein which binds to overlapping SOS boxes, their regulatory sequences including SOS boxes are not identical. Therefore, we investigated at the single cell level, expression of genes encoding three pore formers (A, E1, N), a DNase colicin (E7) and a LPS inhibitor (colicin M), employing *gfp *transcriptional fusions. Our results revealed that, as was previously shown for the *cka *gene [19], only a small portion of the population expressed the investigated activity genes (colicin A, *caa*, Figure 1, Figure 2 and Table 3). We showed that single cell expression of these genes correlates with the predicted affinity of binding of the LexA protein to the operator sequences (Table 3), as expressed by the heterology index (HI). The HI was defined to determine the degree of divergence of any 20 nucleotide sequences from the consensus LexA-binding site [23]. Sequences with a low HI are closer to the consensus and are predicted to bind LexA with greater affinity than sites with a higher HI. Thus, the colicin E7 SOS boxes, which have the highest HI values and therefore the lowest predicted affinity of LexA binding, exhibit approximately three fold higher percentage of cells expressing the colicin activity gene compared to the pore forming colicins examined in this study. On the other hand, single cell analysis of cells harboring a *gfp *fusion with the colicin M activity gene promoter, *cma-gfp*, revealed low level expression in the large majority of the investigated cells. Colicin M was shown to be tightly connected with the upstream colicin B encoding genes and it is presumed that expression of both colicins B and M is regulated from common SOS boxes situated upstream of the colicin B activity gene [16,18]. Colicins M and B are among the most abundant colicins produced by *E. coli *strains [24]. We analysed the nucleotide sequences upstream of *cma *and found neither colicin regulatory motifs nor any consensus promoter sequence (data not presented). Nonetheless, we detected uniform low-level fluorescence mediated by the colicin M promoter (Figure 2, Table 3).

**Table 3:** Cells expressing SOS regulated genes in the wild type RW118

*gfp *transcriptional fusion	% of intensely fluorescent cells	Fluorescence threshold level*	Cell count	HI Distal	Proximal^†^
*caa-gfp *(pSC300)	0.62	41	15555	11.52	9.73

*cna-gfp *(pSC301)	0.51	41	9793	7.55	11.61

*ce1a-gfp *(pSC302)	0.48	41	12197	7.48	11.06

*ce7a-gfp *(pSC303)	1.55	41	9338	12.44	12.98

*recA-gfp *(pSC201)	3.1	57	8366	4.31	

*lexA-gfp *(pSC200)	1.48	57	5089	6.39	8.31

*umuDC-gfp *(pSC202)	0.09	31	2083	2.77	

*Fluorescence threshold level is defined as the point of clear transition from basal level (large majority of cells) to high fluorescence intensity.

^† ^Designated with regard to the ATG codon.

### SOS genes exhibit heterogeneity

Previously, single cell expression of a *sulA-gfp *fusion was investigated [25]. SulA is synthesized in large amounts during the SOS response and inhibits cell division by binding to FtsZ, the major component of the cell division machinery [26]. The *sulA *operator has a HI of 4.65 and thus binds LexA tightly. The authors found that in the absence of exogenous DNA damaging agents only approximately 0.3% of the examined cells fully expressed *sulA*.

**Figure 3:**
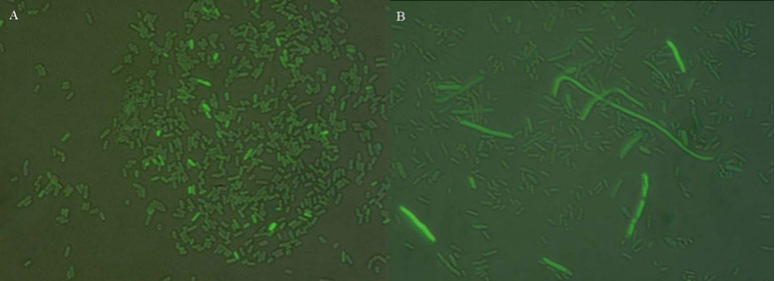
**Merged images of the phase contrast and fluorescence images of *recA-gfp *expression**. **A: **Expression of *recA-gfp *gene fusion in RW118 and **B: **Expression of *recA-gfp *fusion in the isogenic *recA *defective RW464 showing heterogeneity in expression of the *recA *gene.

As RecA is required to initiate the SOS response and LexA to repress the response, both are expressed, albeit at a low level, in the absence of DNA damage. A previous study showed a temporal program of expression of SOS genes upon DNA damage [21]. Subsequently, the response of individual cells to UV irradiation was followed by monitoring the activity of LexA repressed promoters fused to the promoterless *gfp *[27]. The authors found that the response is highly structured as several peaks in promoter activity were observed following DNA damaging UV irradiation. In our study we analyzed at the single cell level, the expression of the *recA*, *lexA*, and *umuDC *genes under physiological conditions using promoter fusions described previously [21]. Fluorescence microscopy revealed heterogeneity in the expression of all three genes. Based on fluorescence intensity, we found that the expression of *recA *(Figure 3) and *lexA *was high in a small percentage of the cells, 3.1 and 1.5%, respectively (Figure 2 and Table 3). In strains harboring the pore formers and DNase colicins transcriptional fusions to the *gfp *gene, heterogeneity was exhibited as a small subpopulation of highly expressing cells within the large majority of non-expressing cells. On the other hand, among the *recA-gfp *and *lexA-gfp *encoding populations, a small fraction exhibited high expression while the large majority exhibited basal level expression. The number of highly fluorescent cells harboring the *recA-gfp *fusion and their fluorescence intensity were higher compared with cells hosting *lexA-gfp*. The HI of the *recA *SOS box is lower than of the *lexA*, predicting a higher affinity of LexA binding however, *lexA *harbors two SOS boxes. These results are in agreement with the higher basal level of the RecA protein compared to LexA, 7,200 versus 1,300 protein molecules per cell, respectively [28]. The higher levels of RecA protein could be explained by its roles in the SOS response, homologous recombination and its involvement in other repair mechanisms such as recombinational repair.

The SOS genes, *polB*, *dinB *and *umuDC*, encode specialized DNA polymerases II, IV and V respectively, which can bypass DNA lesions yet with reduced fidelity introducing mutations to newly synthesized DNA [29]. The operon encoding PolV is regulated by an SOS box that exhibits one of the highest predicted LexA binding affinities, with a HI of 2.77 [30]. It was shown that only upon full induction of the SOS response UmuD is synthesized and persists as a full length dimer [31]. Accordingly, fluorescence emission from the *umuDC-gfp *fusion was observed in a very small fraction of the examined cells (0.09%) and no detectable basal level of expression was observed among the large majority of the population. Our results show that in the absence of exogenous DNA damaging agents very low levels of *umuDC *promoter activity is detected. As translesion synthesis must be employed only when necessary, synthesis of the specialized polymerases is under physiological conditions controlled by complex regulation at the level of transcription and posttranslation.

### SOS regulated genes are expressed in a *recA *defective strain

Besides regulating DNA repair, induction of the SOS response has been recently shown to have a much broader role including, regulation of virulence factor synthesis in *Staphylococcus aureus *[31], type III secretion in enteropathogenic *E. coli *[32], subversion of innate defenses during urinary tract infection [33] and persistence to the fluoroquinolone antibiotic ciprofloxacin [34]. To examine to what extent expression of the investigated pore forming and DNase colicin activity genes as well as the *recA*, *lexA *and *umuDC *genes is dependent upon an SOS response under physiological conditions, expression was investigated in an isogenic *recA *defective strain RW464 (Figure 2). Analysis at the single cell level revealed reduction in the level of fluorescence and the number of intensely expressing cells harboring the *recA, lexA, umuDC, caa *as well as *ce7a *- *gfp *gene fusions and lower fluorescence of cells harboring *ce1a *and *cna*-*gfp *fusions. A greater reduction in the number of intensely expressing cells for colicin A (approximately ten fold) and colicin E7 (approximately three fold) was observed, while the percentage of cells expressing colicin E1 and N activity genes remained essentially unaltered (Figure 2, Table 4). The majority of the *E. coli *LexA regulon promoters are simple, being regulated only by a single transcriptional factor [35] however, some colicin encoding genes have additional regulation. Indeed, the CRP-cAMP complex was shown to stimulate expression of the colicin E1 activity gene *ce1a *by binding upstream from the *ce1a *SOS operator [36,37]. Interestingly, analysis of the colicin N promoter revealed a similar CRP binding site at the same location (Ghazaryan L., personal communication).

**Table 4:** Cells expressing SOS regulated genes in the *recA *defective strain RW464

*gfp *transcriptional fusion	% of intensely fluorescent cells	Fluorescence threshold level*	Cell count
*caa-gfp *(pSC300)	0.075	26	9315

*cna-gfp *(pSC301)	0.45	26	8938

*ce1a-gfp *(pSC302)	0.36	26	7253

*ce7a-gfp *(pSC303)	0.43	26	7050

*recA-gfp *(pSC201)	1.69	52	5695

*lexA-gfp *(pSC200)	0.53	52	2823

*umuDC-gfp *(pSC202)	0.05	24	4004

*Fluorescence threshold level is defined as the point of clear transition from basal level (large majority of cells) to high fluorescence intensity.

Both the *recA-gfp *and *lexA-gfp *fusions were expressed in the *recA *defective strain RW464, albeit at a lower level compared to the wild type (Table 4, Figure 2, Figure 3), with a small fraction of the population exhibiting high fluorescence indicating that, stochastic factors could be involved. Filamentation due to delay in cell division is evident among the less robust *recA *defective strain. However, expression of the investigated genes was not limited to filamented cells (Figure 3).

To resolve the effect of LexA regulation at the single cell level, expression of the investigated gene fusions was also studied in strain RW542 encoding a LexA protein defective in binding to LexA boxes. Fluorescence microscopy revealed that in the *lexA *defective strain all cells harboring the *lexA-gfp *or *recA-gfp *fusions, as well as the large majority (98%) of the cells harboring *gfp *fusions with the colicin activity genes were intensely fluorescent, indicating high level expression (data not shown).

### Simultaneous expression of the *cka *and SOS genes

**Figure 4:**
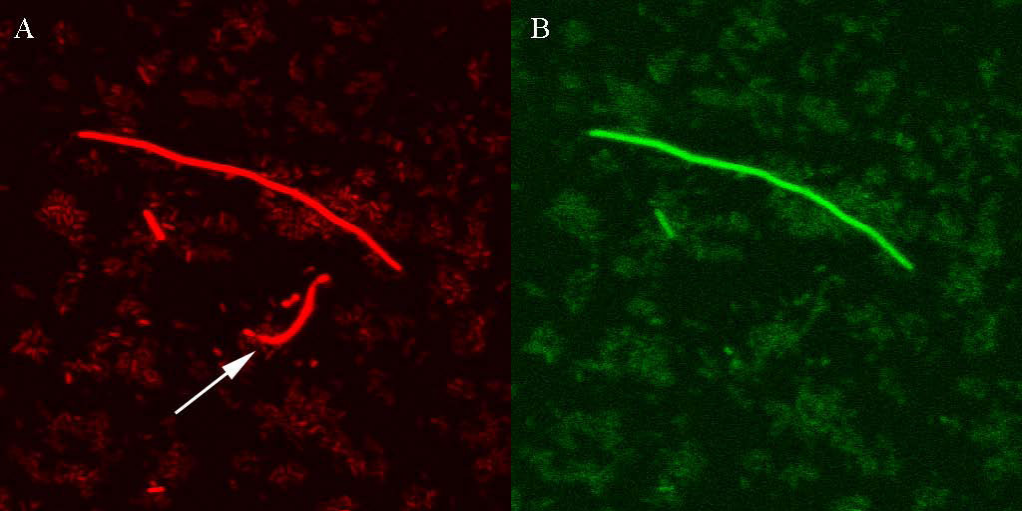
**Fluorescence images showing simultaneous expression of the *cka-DsRed-Express2 *and *lexA-gfp *transcriptional fusions**. **A:**. Expression of *cka-DsRed-Express2 *gene fusion. **B: **Expression of *lexA-gfp *gene fusion in RW118. The arrow indicates a cell with high level expression of *cka*, but not *lexA*, showing that colicin activity gene expression can occur in the absence of the SOS response.

The advent of novel fluorescence markers enables analysis of simultaneous expression of two or more genes. To investigate in detail how the expression of colicin genes correlates with the expression of SOS genes, simultaneous expression of the *cka *and the *lexA *genes was followed at the single cell level in strain RW118 harboring two plasmids: pKCT10 with a *cka-DsRed-Express2 *fusion and the pSC101 derivative vector harboring the *lexA-gfp *fusion. As is evident from Figure 4, the large majority of cells that more highly expressed the *lexA *gene also expressed the *cka *gene. Nonetheless, individual cells (approximately 0.1%) highly expressing only the *cka *gene could be detected suggesting, that in a very small fraction of the population the colicin K activity gene is expressed in the absence of the SOS response most probably stochastically, due to perhaps intracellular fluctuations of the LexA protein. Filamentation while a hallmark of SOS induction due to binding of SulA to the FtsZ proteins is also evident in cells not expressing *lexA-gfp *(Figure 4). Multimers of the natural *cka-gfp *encoding plasmid could be responsible for filamentation in the absence of SOS induction [38].

In conclusion, to our knowledge this is the first study exploring a number of SOS regulated genes at the single cell level under physiological condition. Exposure of a population of bacterial cells to a DNA damaging agent induces the SOS response in all susceptible cells. However, under physiological conditions, genes regulated by the LexA protein also exhibit heterogenous expression. We show that genes with a very high affinity of LexA binding, characteristic of overlapping SOS boxes of colicin operators, or very low HI such as *umuDC*, are expressed in only a small fraction of the population and exhibit no detectable basal level expression. In contrast, genes of the SOS regulon with a somewhat lower predicted affinity of LexA binding, such as *lexA *and *recA*, while also fully expressed in a small subpopulation, exhibit basal level expression. Intense fluorescence of cells harboring the investigated gene fusions was observed in a *lexA *defective strain indicating that the LexA protein effectively represses promoter activity in the large majority of cells. Some of the examined cells could be experiencing disruption of replication forks during replication and thus induction of the SOS response. However, expression of all of the investigated genes was observed in a *recA *mutant, which cannot instigate an SOS response indicating that, expression of LexA regulated genes also occurs stochastically.

Expression of colicin genes under physiological conditions by a small subpopulation may promote strain and genetic diversity and due to lysis of producing cells could provide resources to facilitate growth of non-expressing cells. On the other hand, a subpopulation of cells with higher levels of the RecA protein may be more proficient in recombination, e.g. for the stable incorporation of horizontally acquired DNA or a rapid response to DNA damage. We can speculate that heterogeneity of expression of *lexA *in *E. coli *affects a number of phenomenon significant for antibiotic tolerance/resistance (persisters), horizontal gene transfer (induction of prophage) and virulence among pathogenic *E. coli *strains. The same might apply to other gram negative (e.g. *Shigella*, *Salmonella*, *Pseudomonas aeruginosa*) and gram positive (e.g. *S. aureus*, *B. subtilis*) bacterial species that possess a system similar to the *E. coli *SOS system.

## Conclusion

LexA regulated SOS genes exhibit heterogeneity as they are highly expressed in only a small subpopulation of cells. Unlike *recA *and *lexA*, the colicin activity genes and *umuDC *exhibit no basal level expression. Heterogenous expression is established primarily by stochastic factors as well as the binding affinity of LexA to SOS boxes.

## Authors' contributions

SK performed all experiments. ZP contributed to analysis of the results. OG and DŽB participated in the design of the experiments and SK, OG and DŽB in preparation of the manuscript. All authors read and approved the final manuscript.
